# The Diagnosis Value of a Novel Model with 5 Circulating miRNAs for Liver Fibrosis in Patients with Chronic Hepatitis B

**DOI:** 10.1155/2021/6636947

**Published:** 2021-03-01

**Authors:** Qingqing Zhang, Qidi Zhang, Binghang Li, Ying Qu, Zhenghong Li, Lungen Lu, Rongzhou Li, Xiaobo Cai

**Affiliations:** ^1^Department of Gastroenterology, Ruian People's Hospital, Wenzhou, Zhejiang Province 325200, China; ^2^Department of Gastroenterology, Shanghai General Hospital, Shanghai Jiao Tong University School of Medicine, Shanghai 200080, China

## Abstract

**Methods:**

Differential expression of five selected miRNAs (hsa-mir-1225-3p, hsa-mir-1238, hsa-miR-3162-3P, hsa-miR-4721, and hsa-miR-H7) was verified by qRT-PCR in the plasma of 83 patients and 20 healthy controls. The relative expression of these miRNAs was analyzed in different groups to screen target miRNA. A logistic regression analysis was performed to assess factors associated with fibrosis progression. The receiver operating characteristic (ROC) curve and discriminant analyses validated the ability of these predicted variables to discriminate the nonsignificant liver fibrosis group from the significant liver fibrosis group. Furthermore, the established models were compared with other prediction models to evaluate the diagnostic efficiency.

**Results:**

These five tested miRNAs all had signature correlations with hepatic fibrotic level (*p* < 0.05), and the upregulation trends were consistent with miRNA microarray analysis previously. The multivariate logistic regression analysis identified that a model of five miRNAs (miR-5) had a high diagnostic accuracy in discrimination of different stages of liver fibrosis. The ROC showed that the miR-5 has excellent value in diagnosis of fibrosis, even better than the Forns score, FIB-4, S index, and APRI. GO functions of different miRNAs mainly involved in various biological processes were markedly involved in HBV and revealed signaling pathways dysregulated in liver fibrosis of CHB patients.

**Conclusions:**

It was validated that the combination of these five miRNAs was a new set of promising molecular diagnostic markers for liver fibrosis. The diagnosis model (miR-5) can distinguish significant and nonsignificant liver fibrosis with high sensitivity and specificity.

## 1. Introduction

Hepatitis B virus (HBV) infection has been demonstrated as the major cause of chronic liver diseases which affects about 370 million people in the world [1]. Despite effective vaccination that has resulted in a decrease in acute HBV incidences in many countries, persistent infection of HBV remains a principal challenge [2, 3]. Therefore, it is very important to have an accurate method to diagnose and evaluate chronic hepatitis B- (CHB-) related liver inflammation and fibrosis safely and conveniently [4]. Biopsy was an invasive method with good diagnostic performance with potential risk of various complications, and it is therefore difficult to be performed routinely [5]. However, the values of noninvasive methods such as image examination and blood tests, including fibrosis-related factors, HBV DNA levels, the presence of HBV antigens *2-4*, and aminotransferases (ALT, AST), are limited in distinguishing patients with different fibrotic stages [6–8].

**Figure 1:**
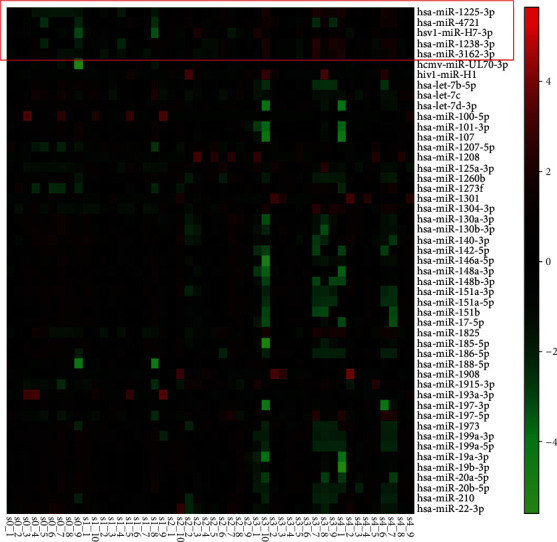
Heatmap of top 50 of 140 different miRNAs in patients with hepatitis B virus infection and hepatic fibrosis.

miRNAs are a class of small noncoding RNAs with ~22 nucleotides that regulate 20~80% of the host genes [9, 10]. A lot of studies have proven that different miRNA expressions are associated with liver fibrosis [11–13]. Wang et al. [14] found that miR-122, miR-194/192, miR-223, miR-21, miR155, and miR-29 were specifically expressed or enriched in several types of hepatic cells or in circulation, playing important roles in the pathogenesis of liver diseases. However, it is difficult to distinguish different stages of CHB by miRNA expression profiles. In our previous study, we found that different expressions of serum/plasma miRNAs are closely associated with hepatic fibrosis staging [15]. We identified 140 detectable miRNAs with fold change of expression ≥ 2 and *p* value < 0.01 in the different groups (Figure 1). We also found that the expressions of hsa-miR-1225-3p, hsv1-miR-H7-3p, hsa-miR-1238-3p, hsa-miR-3162-3p, and hsa-miR-4721 were significantly different between the nonsignificant liver fibrosis group and significant liver fibrosis group. Nevertheless, further investigations are required to explore the diagnostic effects of these 5 miRNA expression profiles.

In this study, these 5 miRNAs were selected as candidates according to our previous study and evaluated in patients with different stages of liver fibrosis. The diagnostic efficiency of our model was also compared with those of other prediction models such as aspartate aminotransferase-to-platelet ratio index (APRI), Forns score, fibrosis index based on the 4 factors (FIB-4), and Spearman correlation (S index). Furthermore, we analyzed the biological function of these miRNAs through bioinformatics analysis. We aim to explore the potential possibility of applying the model including these miRNAs as a stable, reliable, and easy modality for noninvasive diagnosis of hepatic fibrosis.

## 2. Patients and Methods

### 2.1. Patients

The CHB liver fibrosis patient cohort was obtained from Shanghai General Hospital and Chinese PLA No. 85 Hospital from July 2012 to December 2014, and 203 CHB patients were enrolled. The inclusion criteria were based on the diagnostic standards of the CHB set by the Chinese Medical Association: serum HBeAg positive/negative, HBsAg positive, HBV DNA positive, anti-HBe positive or negative, ALT persistent or recurrent abnormality, and histopathological evidence of liver injury. Exclusion criteria were concurrent infection with HIV, HCV, or HDV; alcohol daily intake over 30 g; history of liver transplantation; and liver malignancy, drug-induced liver diseases, autoimmune liver diseases, or other types of liver injury. Liver biopsy pathological examination was performed in all the included patients, and the fibrosis stage was evaluated according to the Scheuer pathological classification system [16]. The component of staging (S) was given in a numerical value ranging from 0 to 4 and used to describe disease progression of chronic HBV infection. S0 represents the absence of fibrosis; S1 represents enlarged, fibrotic portal tracts; S2 represents periportal or portal-portal septa, but intact architecture; S3 represents fibrosis with architectural distortion, but no obvious cirrhosis; and S4 represents definite cirrhosis. The patients with cirrhosis were also divided into Child-Pugh level A (liver compensated cirrhosis) or Child-Pugh level B and Child-Pugh level C (decompensated cirrhosis). Another 20 normal participators aged from 30 to 50 years were enrolled as the control group. Healthy controls were recruited randomly from individuals who had no clinical symptoms of infectious diseases after regular physical examination, and HCV patients enrolled in this study were confirmed to have no other infectious diseases, such as HBV, HIV, and HSV, and have no drug treatment and also have no obvious hepatic steatosis, hepatic fibrosis, and hepatic tumors. This study was approved by the Ethical Committee of Ruian People's Hospital and Shanghai Jiao Tong University Affiliated First People's Hospital, and all the participators voluntarily joined this study and provided tissue specimens with informed consent. Whole blood and biochemical assessment, coagulation function, HBV quantification, and HBV-DNA quantification were performed. Plasma samples were isolated within 1 h after receiving whole blood and then immediately stored at -80°C for standby use.

### 2.2. Taqman PCR Analysis

Total RNA was isolated from patients' plasma using mirVana™ PARIS™ (Ambion, America) following the manufacturer's instructions. The relative expression of miRNA was detected by Taqman PCR. The RNA concentration was checked by using a NanoDrop spectrophotometer and was purified with the QIAGEN Rneasy Mini Kit. After that, total RNA was converted to cDNA using Stem-loop RT primer and TaqMan reverse transcription kit in 15 *μ*L reaction. The PCR was using microRNA assays and PCR Master Mix and run using the Life ViiA™ 7 System thermal cycler with the following settings: 95°C for 10 min and 40 cycles of 10 s at 95°C and 30 s at 60°C. The qPCR data were expressed as minus delta Ct using spike-in control as the reference gene. This experiment adopts the external standard method, and cel-miR-39 was selected as the external reference gene in this experiment.

### 2.3. Assessment of Diagnostic Effectiveness

Diagnostic models of hsa-mir-1225-3p, hsa-mir-1238, hsa-miR-3162-3P, hsa-miR-4721, and hsa-miR-H7 were established, and the diagnostic efficacy was assessed. The receiver operating characteristic curve (ROC curve) of the subjects was established to evaluate the diagnostic efficacy of the individual miRNA, and the area under the curve (AUROC) was calculated by using SPSS 19.0. As a result, the sensitivity and specificity of the differential miRNA and diagnostic models were assessed by the ROC curve and AUROC. At the same time, the diagnostic value of the model was assessed by the ROC curve, and AUROC was compared with the Forns index, APRI, FIB-4 score, and S index of the diagnostic model. The Spearman correlation analysis was used to analyze the correlation between two variables.

### 2.4. Prediction of Target Genes

In order to predict the relative target genes roundly, TargetScan (http://www.targetscan.org/), miRanda (http://www.Microma.org), and RNA22 (http://cbcsrv.watson.ibm.com/rna22.html) were applied to predict the target genes of different miRNAs. In order to reduce the false-positive rate, the genes which were predicted by three software were taken as candidate target genes.

### 2.5. Gene Ontology (GO) Analysis

In order to detect the target genes of those miRNAs with different expression levels and to evaluate their function, GO annotation was applied to the target gene according to the GO database (http://www.geneontology.org/).

### 2.6. Statistical Analysis

The statistical analyses were performed using SPSS 19.0 (IBM, Armonk, NY, USA). Data are presented as the means ± SE. The group *t*-test was used for the data which are consistent with normal distribution, and the Wilcoxon nonparametric test was used for the nonnormal distribution data. *p* values < 0.05 were considered significant. The ROC curve and AUROC analysis were performed using SPSS 19.0 as well. Logistic regression indicates a linear combination of miRNAs. ROC curves and AUROC were used to assess the sensitivity and specificity of miRNA biomarkers and construct the diagnostic models based on the predicted probability. The GO values involved with these genes were determined, and the Fisher exact test and *χ*^2^ test were used to determine the significance level and error rate of each GO value to enable screening of the significant GO terms reflected by the target gene. *p* < 0.01 was considered to indicate a statistically significant difference. In order to determine the cell pathways affected by the target gene with differential miRNA expression, the Fisher exact test and *χ*^2^ square test were used to determine the significance of the pathways, in which the target gene is involved, based on the Kyoto Encyclopedia of Genes and Genomes (KEGG) database. *p* < 0.05 was considered to indicate a statistically significant difference.

## 3. Results

### 3.1. Clinical Characteristics of the Subjects for Hub miRNA Validation

Eighty-three patients with chronic HBV were divided into S0 (10), S1 (15), S2 (13), S3 (10), S4 (10), compensated cirrhosis (13), and decompensated cirrhosis groups (12) according to the Scheuer and Child-Pugh scoring system.  Table 1 shows the demographic and clinical characteristics of all subjects included in the study. At this moment, CHB liver fibrosis patients were classified into two groups: nonsignificant liver fibrosis group and significant liver fibrosis group. The nonsignificant liver fibrosis group consisted of 10 S0 and 15 S1 cases, while the significant liver fibrosis group consisted of 13 S2, 10 S3, and 10 S4 cases. Blood platelet differed significantly between the nonsignificant liver fibrosis group and significant liver fibrosis group (*p* < 0.05, Table 2). In order to know the difference of routine clinical indices between liver fibrosis patients and cirrhosis patients, the subjects were divided into the liver fibrosis group (consisting of S0-S3) and cirrhosis group (consisting of S4, compensated cirrhosis, and decompensated cirrhosis groups). And there were statistically different laboratory results for age, hemoglobin, blood cell counts, blood platelet, AST, TG, TC, AFP, INR, and HBeAg-positive rate between the two groups (*p* < 0.05, Table 3). In addition, there were significant differences of AST, ALP, ALB, INR, T-Bil, and D-Bil between compensated cirrhosis patients (*p* < 0.05, Table 4).

### 3.2. mRNA Expression Validation of Hub miRNAs in Participators

**Figure 2:**
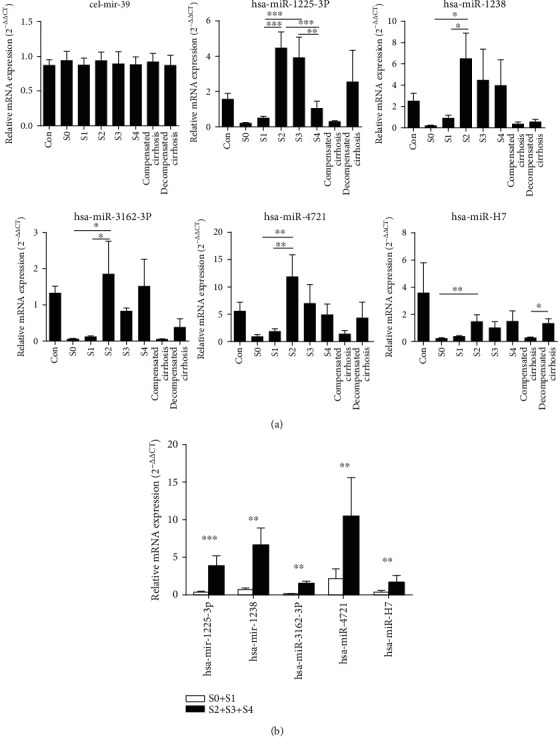
Relative mRNA expression level of different plasma microRNAs in cases of the chronic hepatitis B group and control group. (a) mRNA expression of hsa-mir-1225-3p, hsa-mir-1238, hsa-miR-3162-3P, hsa-miR-4721, hsa-miR-H7, and cel-mir-39 in normal control; CHB liver fibrosis S0 patients, S1 patients, S2 patients, S3 patients, and S4 patients; and CHB compensated cirrhosis patients and CHB decompensated cirrhosis patients. (b) mRNA expression of hsa-mir-1225-3p, hsa-mir-1238, hsa-miR-3162-3P, hsa-miR-4721, hsa-miR-H7, and cel-mir-39 in the CHB nonsignificant liver fibrosis group (S0-1) and significant liver fibrosis group (S2-4).

**Figure 3:**
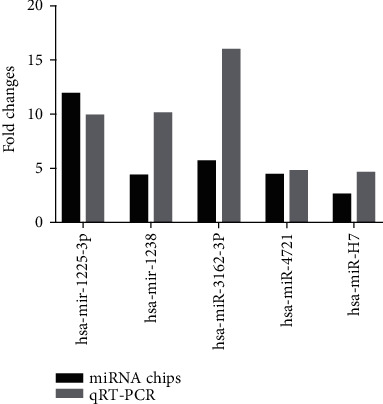
The fold changes of miRNA expression in the CHB nonsignificant liver fibrosis group and significant liver fibrosis group and their comparison in miRNA chips.

According to our previous study, 5 miRNAs include hsa-mir-1225-3p, hsa-mir-1238, hsa-miR-3162-3P, hsa-miR-4721, and hsa-miR-H7 which were chosen for further investigation. The expression of the 5 miRNAs was analyzed between the different groups. As shown in Figure 2(a), the expression level of the 5 miRNAs was significantly different between the nonsignificant liver fibrosis group and significant liver fibrosis group (*p* < 0.05). At the same time, the expressions of hsa-mir-1225-3p, hsa-mir-1238, hsa-miR-3162-3p, hsa-miR-4721, and hsa-miR-H7 were upregulated more than 2-fold in the mild-severe fibrosis group (S0-1) and significant liver fibrosis group (S2-4). The trend of miRNA expression is consistent with the chip results, although the upregulation extent is not completely consistent with the chip results, and the results showed great reliability and credibility (Figures 2(b) and 3). Furthermore, single target miRNA of hsa-mir-1225-3p, hsa-mir-1238, hsa-miR-3162-3P, hsa-miR-4721, and hsa-miR-H7 cannot distinguish liver fibrosis (S0-3) from cirrhosis and compensated cirrhosis from decompensated cirrhosis (Table 5).

### 3.3. Diagnosis Value of Hub miRNAs for CHB Liver Fibrosis and Diagnostic Model Establishment

**Figure 4:**
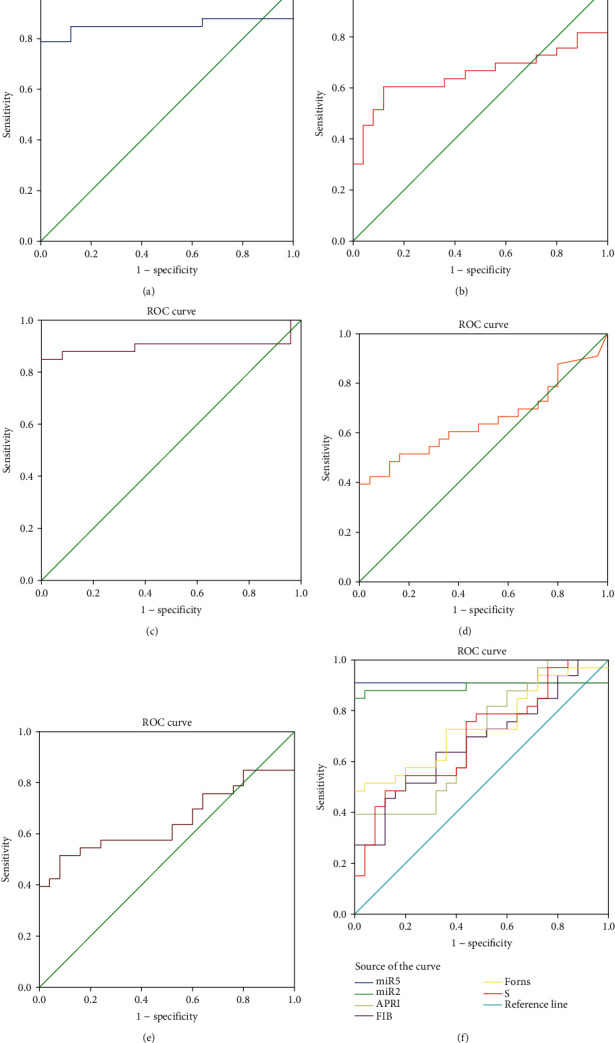
ROC curve analysis. ROC curve of hsa-mir-1225-3p (a), hsa-mir-1238 (b), hsa-miR-3162-3P (c), hsa-miR-4721 (d), and hsa-miR-H7 (e) in predicting significant liver fibrosis (S2-4). (f) ROC curve of diagnostic models miR-5, miR-2, APRI, FIB-4, Forns, and S in predicting significant liver fibrosis (S2-4).

To explore the diagnostic potential of verified miRNAs for liver fibrosis in CHB patients, ROC curves were constructed. When the target miRNAs were used to diagnose significant hepatic fibrosis, the AUROC for hsa-miR-3162, hsa-miR-1225, hsa-miR-1238, hsa-miR-4721, and hsa-miR-H7 were as follows: 0.899 (95% CI: 0.803-0.995), 0.852 (95% CI: 0.737-0.967), 0.656 (95% CI: 0.521-0.803), 0.650 (95% CI: 0.508-0.792), and 0.650 (95% CI: 0.506-0.794), respectively. The results showed that miRNA-3162 and miRNA-1225 had relatively higher accuracy in indicating hepatic fibrosis than hsa-miR-1238, hsa-miR-H7, and hsa-miR-4721 (Figures 4(a)–4(e), Table 6). And then, two forecasting models of miR-5 (hsa-miR-3162, hsa-miR-1225, hsa-miR-1238, hsa-miR-4721, and hsa-miR-H7) and miR-2 (miRNA-3162 and miRNA-1225) were obtained from logistic regression analysis to predict significant hepatic fibrosis (S2-S4). The miR-5 and miR-2 were miR‐5 = 1.115∗miR1225 + 0.157∗miR1238 + 6.256∗miR3162 + 0.072∗miR4721 − 0.831∗miRH7 − 2.729 and miR‐2 = 0.483∗miR1225 + 7.066∗miR1238 − 2.276, respectively. To evaluate whether miR-5 and miR-2 can be used as potential diagnostic models for significant hepatic fibrosis, ROC curve analyses were performed. The value of AUROC for miR-5 and miR-2 was 0.909 and 0.895, respectively, and revealed that miR-5 was superior to miR-2 in discriminating the significant hepatic fibrosis group (S2-S4) from the nonsignificant liver fibrosis group (S0-S1) (Figure 4(f), Table 6).

### 3.4. Comparison of Diagnostic Efficiency between miR-5 and Other Diagnostic Models

Noninvasive diagnosis indices or predictive models for hepatic fibrosis, including the Forns index, APRI, and FIB-4, are all established based on CHB. To analyze the correlation between each diagnostic model and liver fibrosis, the correlation coefficients (*r*) of the five miRNAs and five diagnostic models were evaluated by the Spearman correlation. The *r* values of miRNA-1225, miRNA-3162, miRNA-H7 and miR-5 were 0.267, 0.270, 0.295, and 0.632, respectively (*F* < 0.05). There was no statistical significance for miRNA-1238, miRNA-4721, and APRI for liver fibrosis stages in these subjects. In the prediction of significant hepatic fibrosis (S2-S4), the value of AUROC for these miRNAs and diagnostic models is shown in Figure 4(f); the diagnostic models of miR-5, miRNA-3162, miRNA-1225, and Forns index had high diagnostic value; and miR-5 had the highest AUROC and the highest diagnostic efficiency.

To further evaluate the diagnostic efficacy of the model miR-5 for significant liver fibrosis (S2-S4), the AUROC of these diagnostic models and five miRNAs were analyzed by using the MedCalc software. The result showed that the diagnostic efficacy of miR-5 was significantly better than that of the Forns index, APRI, FIB-4 score, S index, miRNA-H7, miRNA-4721, and miRNA-1238 (*p* < 0.05). At the same time, at a cut-off value of 0.37 for miR-5, the sensitivity was 90.6% and the specificity was 96% in discriminating significant liver fibrosis (Table 7).

### 3.5. Bioinformatics Analysis

GO functional analysis of 105 differential miRNAs (Table 3) revealed that various biological processes were markedly involved in HBV-related liver fibrosis including biopolymer metabolic processes, signal transduction, protein metabolism, and lipid metabolism. And the same result was obtained from the GO functional analysis of 5 differential miRNAs. The biological processes which were identified to have significant involvement of the differently expressed target gene included molecule functional activation, transmembrane transport, phosphotransferase activity upgrade, binding with protein, purine nucleotide metabolism upgrade, peptidase activation, oxidoreductase activation, binding with L-amino acid peptidase, and cytokine activation.

**Figure 5:**
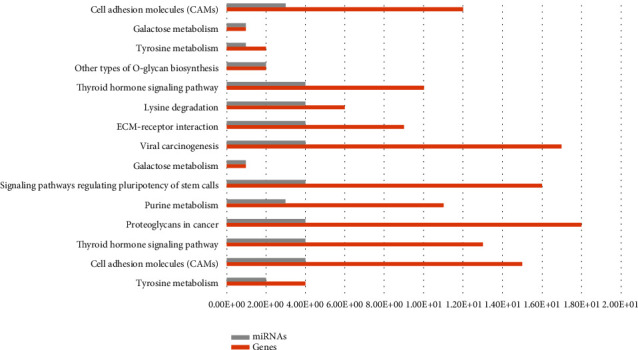
Functional enrichment KEGG pathway analysis of miRNAs.

Pathway significance concentration analysis revealed that the differentially expressed genes were involved in 100 significant signal transduction pathways, including TGF-*β*/Smad, Wnt, MAPK, Jak/STAT, and VEGF, which have a significant impact on hepatic fibrosis (Figure 5).

## 4. Discussion

Early diagnosis and sustained follow-up of liver fibrosis are essential for the prevention of liver cirrhosis and end-stage hepatic disease [17]. Liver biopsy is an invasive procedure associated with complications and limitations such as intraobserver and interobserver variation, sampling error, and variability [18]. Therefore, many studies and great interest have been dedicated to the development of noninvasive tests to substitute liver biopsy for fibrosis assessment and follow-up [19, 20]. There are lots of noninvasive diagnosis indices and predictive models for hepatic fibrosis based on chronic hepatitis C (CHC) and alcoholic liver diseases, such as the Forns index [21], FibroTest [22], and APRI [23, 24]. However, a specific noninvasive predictive model for chronic HBV infection and hepatic fibrosis has not yet been developed.

miRNAs with high stability are good noninvasive diagnostic and prognostic markers to predict and monitor a disease in circulation [25]. Circulating miRNAs as diagnostic markers for different cancers were extensively studied in recent years. It has been verified that miRNAs play critical functional roles in HBV-driven hepatocellular carcinoma (HCC) [26–28]. However, there are controversies about the origin of circulating miRNAs and lack of specificity to reflect the disease biology. miR-144 is such a marker which has been identified as a circulating biomarker for different cancers such as hepatocellular carcinoma, renal cell carcinoma, and oral squamous cell carcinoma [29], whereas 6 serum miRNAs (miR-21, miR-199a-5p, miR-200c, miR-31, let-7a, and let-7d) have been identified as biomarkers for idiopathic pulmonary fibrosis, which still needs further validation [30, 31]. However, miRNAs for distinguishing different stages of liver fibrosis have not been well studied. During different statuses of CHB, the profiling of miRNA is also important for understanding the mechanisms of HBV-driven disease progression [2].

In our previous study, there were significant correlations between the levels of five miRNAs (hsa-mir-1225-3p, hsa-mir-1238, hsa-miR-3162-3P, hsa-miR-4721, and hsa-miR-H7) and CHB with different liver fibrosis stages [15]. In this study, we further identified these 5 miRNAs with distinct expression profiles in CHB patients with liver fibrosis. Statistical results showed that the level of clinical indicators did not correlate with the liver biopsy result well. The levels of target miRNAs were significantly different between the nonsignificant and significant liver fibrosis groups. The diagnosis effectiveness of the identified miRNAs in distinguishing fibrosis stages in CHB patients was determined by ROC curve analysis, whereas most of the previous studies have compared only healthy controls with CHB patients [32]. In this study, 2 logistic diagnosis models (miR-5 and miR-2) with different miRNAs were established, and our results demonstrate that miR-5 performed better than other diagnosis indices and predictive models (Forns index, S index, FIB-4, and individual miRNAs) in distinguishing different liver fibrosis stages.

CHB patients could develop hepatic decompensation and related complications [33]. To evaluate the relationship between the miRNA markers and the compensatory capability of liver function, we examined the levels of the miRNAs in compensated or decompensated cirrhosis patients. We found that the 5 miRNAs were upregulated as deterioration of liver compensatory ability and significantly different between the two groups. Therefore, these miRNAs can be used to evaluate liver decompensation in addition to diagnosing the stage of liver fibrosis. At the same time, the optimum cut-off level of 0.37 of miR-5 was also determined for the diagnosis of liver fibrosis in CHB patients with the sensitivity of 90.6% and specificity of 96% in discriminating different stages of CHB fibrosis.

Genome informatics analysis also indicates that liver fibrosis is related to the GO functions of these differential miRNAs associated with biological processes, such as biopolymers, signal transduction, protein metabolism, and lipid metabolism. The pathway significance analysis revealed more than 100 pathways, which were significantly dysregulated in human CHB-related liver fibrosis. All of these genomic phenomena will help us to explore the potential pathogenesis of this disease.

In conclusion, our study emphasizes the importance of an integrated approach to identify a useful circulating biomarker for the diagnosis of the disease. A comparatively larger sample size might improve the strength of the study, but our findings elucidate a correlation of these disease-specific hepatic miRNAs as biomarkers in detecting human CHB liver fibrosis. Further analysis of the expression profiles of the miRNAs in different stages of liver fibrosis revealed that these miRNAs were differently expressed in certain stages of fibrosis. All these findings would benefit our understanding of the expression profiles of circulating miRNAs in different stages of HBV-driven disease and the development of novel noninvasive diagnostic tools for the identification of fibrosis in CHB patients. In addition, other categorization factors such as inflammation and qualification of viral load will be explored in the further study.

## Figures and Tables

**Table 1:** Clinical characteristics of the 83 participators ($\bar{x}\pm s$).

Clinical characteristics	Control group (*n* = 20)	S0 group (*n* = 10)	S1 group (*n* = 15)	S2 group (*n* = 13)	S3 group (*n* = 10)	S4 group (*n* = 10)	CC group (*n* = 13)	DC group (*n* = 12)
Male/female	10/10	6/4	10/5	8/5	6/4	7/3	9/4	8/4
Age (years old)	38.8 ± 9.8	40.7 ± 14.9	33.8 ± 9.4	38.0 ± 8.4	39.4 ± 13.2	50.1 ± 12.6	51.3 ± 13.1	50.2 ± 17.4
Blood cell counts (10^9^/L)	7.3 ± 2.1	5.9 ± 0.6	5.8 ± 1.0	5.8 ± 3.4	4.3 ± 1.2	5.8 ± 3.4	5.0 ± 1.8	4.2 ± 1.8
Hemoglobin (g/L)	140 ± 12	151 ± 15	148 ± 23	144 ± 16	136 ± 32	144 ± 19	130 ± 31	90 ± 19
Blood platelet (10^9^/L)	175 ± 57	173 ± 12.8	175 ± 12.1	158 ± 24.1	121.3 ± 15.4	154.3 ± 20.1	105.3 ± 16.7	70.2 ± 17.8
ALT (U/L)	26.3 ± 9.3	79.5 ± 42.0	95.3 ± 33.3	124.1 ± 29.0	91.7 ± 17.5	57.1 ± 14.3	91.8 ± 15.2	30.8 ± 10.1
AST (U/L)	23.5 ± 7.1	65.2 ± 23.8	69.4 ± 11.2	91.6 ± 21.5	92.75 ± 19.9	46.4 ± 19.7	67.2 ± 17.2	56.1 ± 12.6
*γ*-GT (U/L)	31.2 ± 5.7	37.0 ± 18.1	50.8 ± 38.4	76.9 ± 25.8	58.8 ± 28.3	63.2 ± 29.1	61.1 ± 19.2	37.9 ± 13.4
ALP (U/L)	68.0 ± 11.0	69.2 ± 24.4	79.8 ± 22.5	125.5 ± 46.8	94.6 ± 27.9	87.9 ± 11.6	83.6 ± 13.2	100 ± 15.5
T-Bil (*μ*mol/L)	13.6 ± 5.3	27.9 ± 7.41	22.2 ± 8.04	26.9 ± 3.2	19.1 ± 2.3	12.9 ± 4.4	23.3 ± 8.3	68.6 ± 12.3
D-Bil (*μ*mol/L)	5.0 ± 1.2	15.2 ± 5.0	6.5 ± 2.2	11.6 ± 6.1	8.2 ± 3.7	3.6 ± 1.1	7.5 ± 3.3	47.9 ± 9.1
TP (g/L)	74.6 ± 8.3	67.6 ± 8.8	68.6 ± 8.3	72.4 ± 6.2	68.7 ± 8.0	66.8 ± 7.5	66.3 ± 9.6	53.2 ± 5.8
ALB (g/L)	44.5 ± 3.2	40.7 ± 3.7	40.2 ± 4.7	41.5 ± 3.6	39.0 ± 5.1	39.1 ± 5.1	30.3 ± 8.6	22.3 ± 9.4
TC (U/L)	3.3 ± 0.4	4.4 ± 1.0	3.2 ± 0.3	3.6 ± 0.4	3.8 ± 1.1	3.6 ± 0.8	4.8 ± 1.2	3.4 ± 0.2
TG (U/L)	0.6 ± 0.1	1.0 ± 0.4	1.0 ± 0.1	1.7 ± 0.4	1.2 ± 0.4	1.1 ± 0.4	1.4 ± 0.2	1.3 ± 0.3
AFP	12 ± 1.2	18.7 ± 32.1	6.2 ± 4.1	8.0 ± 13.4	28.8 ± 34.8	99.2 ± 12.1	104.2 ± 11.1	31.5 ± 11.2
INR	0.8 ± 0.01	0.97 ± 0.09	1.0 ± 0.03	1.1 ± 0.08	1.05 ± 0.07	1.6 ± 0.06	1.7 ± 0.02	1.8 ± 0.08
HBV-DNA (10^7^ U/L)	—	4.5 ± 1.1	13.1 ± 2.7	5.2 ± 2.1	3.1 ± 1.1	1.3 ± 0.2	0.9 ± 0.2	0.6 ± 0.3
HBeAg-positive rate (%)	—	30%	80%	58%	60%	40%	38%	33%

CC: compensated cirrhosis; DC: decompensated cirrhosis; CHB: chronic hepatitis B.

**Table 2:** Clinical characteristics of the CHB nonsignificant liver fibrosis group (S0-1) and significant liver fibrosis (S2-4) cases ($\bar{x}\pm s$).

Clinical characteristics	S0-1	S2-4	*p*
Male/female	16/9	21/12	1.0
Age (years old)	35.4 ± 14	42 ± 12	0.064
Blood cell counts (10^9^/L)	5.8 ± 0.9	5 ± 0.4	0.118
Hemoglobin (g/L)	149 ± 22	142 ± 23	0.224
Blood platelet (10^9^/L)	173 ± 20	145 ± 17	0.04
ALT (U/L)	60.1 ± 12.3	94 ± 11.2	0.107
AST (U/L)	51.2 ± 7.4	78 ± 6.3	0.109
*γ*-GT (U/L)	45.2 ± 13.9	67 ± 15	0.123
ALP (U/L)	73.4 ± 7.7	104 ± 14.1	0.09
T-Bil (*μ*mol/L)	22.2 ± 8.9	32.2 ± 7.2	0.761
D-Bil (*μ*mol/L)	8.3 ± 5.4	15.3 ± 3.9	0.807
TP (g/L)	64.8 ± 13	69.6 ± 7.3	0.148
ALB (g/L)	36.8 ± 16.2	32 ± 5.9	0.291
TC (U/L)	4.3 ± 0.7	4.4 ± 1.3	0.889
TG (U/L)	1.47 ± 0.7	1.1 ± 0.3	0.065
AFP	10.8 ± 1.7	41.9 ± 8.3	0.08
INR	0.9 ± 0.3	1.1 ± 0.02	0.233
HBV-DNA (10^7^ U/L)	9.8 ± 0.9	4.8 ± 0.6	0.743
HBeAg-positive rate (%)	60%	72%	0.399

**Table 3:** Clinical characteristics of the CHB liver fibrosis and liver cirrhosis group cases ($\bar{x}\pm s$).

Clinical data	Liver fibrosis group	Liver cirrhosis group	*p*
Male/female	21/11	30/18	0.815
Age (years old)	38 ± 12	52 ± 11	0.0001
Blood cell counts (10^9^/L)	5.6 ± 2.0	4.6 ± 1.6	0.025
Hemoglobin (g/L)	145 ± 23	124 ± 28	0.001
Blood platelet (10^9^/L)	155 ± 12	112 ± 15.5	0.002
ALT (U/L)	84 ± 16.3	60 ± 21.2	0.285
AST (U/L)	71 ± 21	39 ± 11.2	0.023
*γ*-GT (U/L)	54 ± 12.1	53 ± 31.2	0.926
ALP (U/L)	90 ± 32.1	90 ± 17.3	0.985
T-Bil (*μ*mol/L)	22 ± 12.1	35 ± 13.2	0.160
D-Bil (*μ*mol/L)	9.9 ± 1.8	19.1 ± 9.5	0.177
TP (g/L)	68 ± 13	65 ± 8.3	0.244
ALB (g/L)	39 ± 9.6	35 ± 6.7	0.104
TC (U/L)	4.5 ± 1.1	3.7 ± 0.8	0.004
TG (U/L)	1.3 ± 0.6	0.9 ± 0.3	0.003
AFP	14 ± 11.1	45 ± 8.5	0.029
INR	1 ± 0.2	1.4 ± 0.6	0.001
HBV-DNA (10^7^ U/L)	8.7 ± 0.6	6.3 ± 0.7	0.932
HBeAg-positive rate (%)^∗^	95%	45%	0.0001

**Table 4:** Clinical characteristics of the CHB liver cirrhosis CC and DC group cases ($\bar{x}\pm s$).

Clinical data	CC group	DC group	*p*
Male/female	16/7	8/4	0.071
Age (years old)	50.8 ± 13.11	54.4 ± 7.39	0.388
Blood cell counts (10^9^/L)	4.8 ± 1.57	4.2 ± 1.87	0.430
Hemoglobin (g/L)	135 ± 27	107 ± 19	0.005
Blood platelet (10^9^/L)	126 ± 26	85 ± 11	0.109
ALT (U/L)	76 ± 9.7	30 ± 9.4	0.181
AST (U/L)	58 ± 13	3.7 ± 0.64	0.0001
*γ*-GT (U/L)	62 ± 12	37 ± 7.5	0.086
ALP (U/L)	85 ± 12.6	100 ± 34.2	0.405
T-Bil (*μ*mol/L)	18 ± 8.5	68 ± 7.5	0.043
D-Bil (*μ*mol/L)	5.8 ± 3.3	44 ± 7.9	0.031
TP (g/L)	66 ± 8.6	62 ± 7.3	0.164
ALB (g/L)	37 ± 7.3	32 ± 4.4	0.050
TC (U/L)	3.8 ± 1.0	3.7 ± 0.6	0.775
TG (U/L)	1.0 ± 0.3	0.72 ± 0.24	0.001
AFP	52 ± 12	31 ± 9.3	0.459
INR	1.17 ± 0.21	1.91 ± 0.87	0.015
HBV-DNA (10^7^ U/L)	9.8 ± 0.9	5.3 ± 0.7	0.782
HBeAg-positive rate (%)^∗^	60%	33%	0.164

**Table 5:** *p* values of miRNA expression in the CHB liver fibrosis group, liver cirrhosis group, compensated cirrhosis group, and decompensated cirrhosis group.

miRNA	S0-S3 vs. liver cirrhosis		CC vs. DC	
	*t*	*p*	*t*	*p*
hsa-mir-1225-3p	1.277	0.205	-1.066	0.309
hsa-mir-1238	1.284	0.203	0.892	0.379
hsa-miR-3162-3P	0.386	0.701	0.602	0.551
hsa-miR-4721	1.112	0.269	-0.459	0.649
hsa-miR-H7	-0.712	0.478	-0.966	0.341

**Table 6:** Analysis of the AUROC between the miRNA-5 diagnosis model and other diagnosis models.

Variable	Area	SE	*p* value	Sensitivity (%)	Specificity (%)	95% CI	
						Lower	Upper
miR-5	0.909	0.050	<0.001	90.9	100	0.811	1.000
miRNA-3162	0.899	0.049	<0.001	84.8	100	0.803	0.995
miR-2	0.895	0.051	<0.001	84.8	100	0.794	0.995
miRNA-1225	0.852	0.059	<0.001	78.8	100	0.737	0.967
S	0.697	0.069	0.011	48.5	88	0.562	0.832
APRI	0.696	0.069	0.011	81.8	48	0.560	0.832
miRNA-1238	0.662	0.072	0.036	60.6	80	0.521	0.803
Forns	0.737	0.065	0.002	48.5	100	0.610	0.864
miRNA-4721	0.650	0.073	0.052	39.4	100	0.508	0.792
miRNA-H7	0.650	0.073	0.052	51.5	92	0.506	0.794
FIB-4	0.679	0.070	0.021	45.5	88	0.542	0.815

**Table 7:** Clinical characteristics of the 57 chronic hepatitis B patients for miRNA chip analysis ($\bar{x}\pm s$).

Group	Critical value		Se	Spe	PPV	NPV	J	LR+	LR-	AUROC
	<0.37	≥0.37								
S0-1	29	1	90.6%	96%	96.6%	88.8%	0.866	22.65	0.097	0.909
S2-4	3	24								

## Data Availability

The data used to support the findings of this study are included within the article.
